# The effectiveness of a chair intervention in the workplace to reduce musculoskeletal symptoms. A systematic review

**DOI:** 10.1186/1471-2474-13-145

**Published:** 2012-08-13

**Authors:** Sjan-Mari van Niekerk, Quinette Abigail Louw, Susan Hillier

**Affiliations:** 1 Department of Physiotherapy, Interdisciplinary Health Sciences, Faculty of Health Sciences, Stellenbosch University, P O Box 19063, Tygerberg, 7505, South Africa; 2 International Centre for Allied Health Evidence, School of Health Sciences, University of South Australia, GPO Box 2471, Adelaide, 5000, Australia

## Abstract

**Background:**

Prolonged sitting has been associated with musculoskeletal dysfunction. For desk workers, workstation modifications frequently address the work surface and chair. Chairs which can prevent abnormal strain of the neuromuscular system may aid in preventing musculo-skeletal pain and discomfort. Anecdotally, adjustability of the seat height and the seat pan depth to match the anthropometrics of the user is the most commonly recommended intervention. Within the constraints of the current economic climate, employers demand evidence for the benefits attributed to an investment in altering workstations, *however this evidence-base is currently unclear both in terms of the strength of the evidence and the nature of the chair features*. *The purpose of this study was to evaluate the evidence for the effectiveness of chair interventions in reducing workplace musculoskeletal symptoms.*

**Methods:**

Pubmed, Cinahl, Pedro, ProQuest, SCOPUS and PhysioFocus were searched. ‘Ergonomic intervention’, ‘chair’, ‘musculoskeletal symptoms’, ‘ergonomics’, ‘seated work’ were used in all the databases. Articles were included if they investigated the influence of chair modifications as an intervention; participants were in predominantly seated occupations; employed a pre/post design (with or without control or randomising) and if the outcome measure included neuro-musculoskeletal comfort and/or postural alignment. The risk of bias was assessed using a tool based on *The Cochrane Handbook*.

**Results:**

Five studies were included in the review. The number of participants varied from 4 to 293 participants. Three of the five studies were Randomised Controlled Trials, one pre and post-test study was conducted and one single case, multiple baselines (ABAB) study was done. Three studies were conducted in a garment factory, one in an office environment and one with university students. All five studies found a reduction in *self-reported* musculoskeletal pain *immediately* after the intervention. Bias was introduced due to poor randomization procedures and lack of concealed allocation. Meta-analysis was not possible due to the heterogeneity of the data (differing population, intervention and outcomes across studies).

**Conclusion:**

The findings of this review indicate a consistent trend that supports the role of a chair intervention to reduce musculoskeletal symptoms among workers who are required to sit for prolonged periods. However the amount, level and quality of the evidence are only moderate therefore we cannot make strong recommendations until further trials are conducted. The review also highlights gaps: for example in showing whether the effectiveness of a chair intervention has long-term impact, particularly with respect to musculoskeletal symptoms, as well as the recurrence of symptoms and the consequent cost of care.

## Background

Prolonged sitting at sub-optimal workstations is associated with musculoskeletal dysfunction [1-5]. The musculoskeletal dysfunction presents as pain or muscle tension of the cervical, shoulder, and lumbar regions. A range of modifiable and non-modifiable risk factors are associated with the musculoskeletal symptoms. Non-modifiable risk factors are genetic predisposition, structural spinal deformities or disorders and female gender. The modifiable factors include body alignment (posture), nature and duration of the tasks and job demands as well as physical features of the work [5,6]. Commitment from supervisors and employees is essential to modify these risk factors; so is capital investment to improve the ergonomic design of workstations in an attempt to reduce the occurrence of musculoskeletal symptoms.

Workstation modifications frequently address the work surface and chair [5,7-10]. Since the chair has a direct influence on body alignment (posture), individuals suffering from musculoskeletal symptoms related to prolonged sitting are often advised to alter the chair of their workstations [5,7-10]. Changing the chair is also the most pragmatic action because altering the work surface may be limited by physical space constraints and an adjustable work surface is not always economically viable. Therefore modifying the workstation’s chair is often the most feasible initial step to ascertain whether the design of the workstation is associated with the musculoskeletal symptoms.

In the selection of a chair, adjustability of the seat height and the seat pan depth in correlation with the anthropometrics of the user should be taken into consideration [11,12]. A mismatch in the dimensions of the chair impairs the ability of the postural muscles to support the body and could also lead to abnormal strain of the neuromuscular system, consequently causing pain [5,13,14]. Chairs which can prevent these effects can thus be beneficial in the prevention of spinal pain. A chair meeting the ergonomic requirements is thus postulated to reduce the occurrence of musculoskeletal symptoms.

Musculoskeletal dysfunction in the workplace is typically classified as repetitive strain disorders, which account for about one third of the related injuries leading to absenteeism [15]. The loss of productivity amounts to about $3.3 billion per annum in Washington State, USA [10]. Within the constraints of the current economic climate, employers demand evidence for the benefits attributed to an investment to alter workstations. Therefore, the aim of this study is to appraise the evidence base for the effectiveness of a chair intervention in the workplace to reduce musculoskeletal symptoms.

## Methods

### Search strategy

The following medical electronic databases were searched between inception of the research to March 2011: Pubmed, Cinahl, Pedro, ProQuest, SCOPUS and PhysioFocus. The same search terms, ‘ergonomic intervention’, ‘chair’, ‘musculoskeletal symptoms’, ‘ergonomics’, ‘seat*’, ‘work*’, were used in all the databases *with the appropriate truncations and Boolean operators (such as AND and OR). The search terms were selected using an iterative process of maximising yield and were based on the population (ergonomic, seated, workers) and the intervention (chair, ergonomic) with a broad outcome (musculoskeletal) in line with standard search criteria. Pearling (checking the reference lists of identified studies) and hand searching (journals predating electronic databases or not appearing in electronic databases) were also conducted to increase the search base.* Two reviewers (SH and SvN) independently screened the selected titles and abstracts for eligibility, whilst a third reviewer was available if disagreement arose (QL).

### Inclusion criteria

Articles were deemed eligible if they met all the following inclusion criteria:

Studies which postulate that the chair has an influence on biomechanics;

Studies with children or adults in predominantly seated occupations;

Any trial with pre and post testing, including controlled, randomised or a single subject design;

The outcome measure included neuro-musculoskeletal comfort and/or postural alignment. *Examples of these outcomes include (but are not limited to) signs and symptoms of pain and discomfort that may be attributable to biomechanical alterations of the neuro-musculo-skeletal systems, as distinct from pain from an alternative pathology such as systemic joint disease.*

No date restrictions were applied and only English articles were included because of lack of access to translation services.

Full-text articles were retrieved for those studies that appeared to meet the inclusion and exclusion criteria, and for those in which insufficient information was presented in the title, abstract and key words to determine eligibility.

### Risk of bias assessment

The risk of bias in the selected studies was assessed using 6 criteria recommended by the Cochrane Back Review Group and based on *The Cochrane Handbook*[16]. The criteria were scored ‘yes’, ‘no’ or ‘unclear’ and are reported in the *Risk of Bias* tables. A trial with low risk of bias was defined as a trial that met, at a minimum, criteria 1 (randomisation), 2 (allocation concealment), 5 (outcome assessor blinding) and any three of the other criteria. Two review authors (SvN, SH) independently assessed a selection of trials for risk of bias and reached consensus on the final results. A third review author (QL) assessed the risk of bias for all included studies.

### Data extraction

One reviewer (SH) extracted the data by using a standard data-extraction form. Information on study design, population and outcomes was extracted. If data were missing, first authors of the studies were contacted and additional information was requested. A second reviewer (SvN) audited data extraction accuracy. *The third author was available to facilitate consensus if there was a disagreement.*

## Results

### Study selection

**Figure 1:**
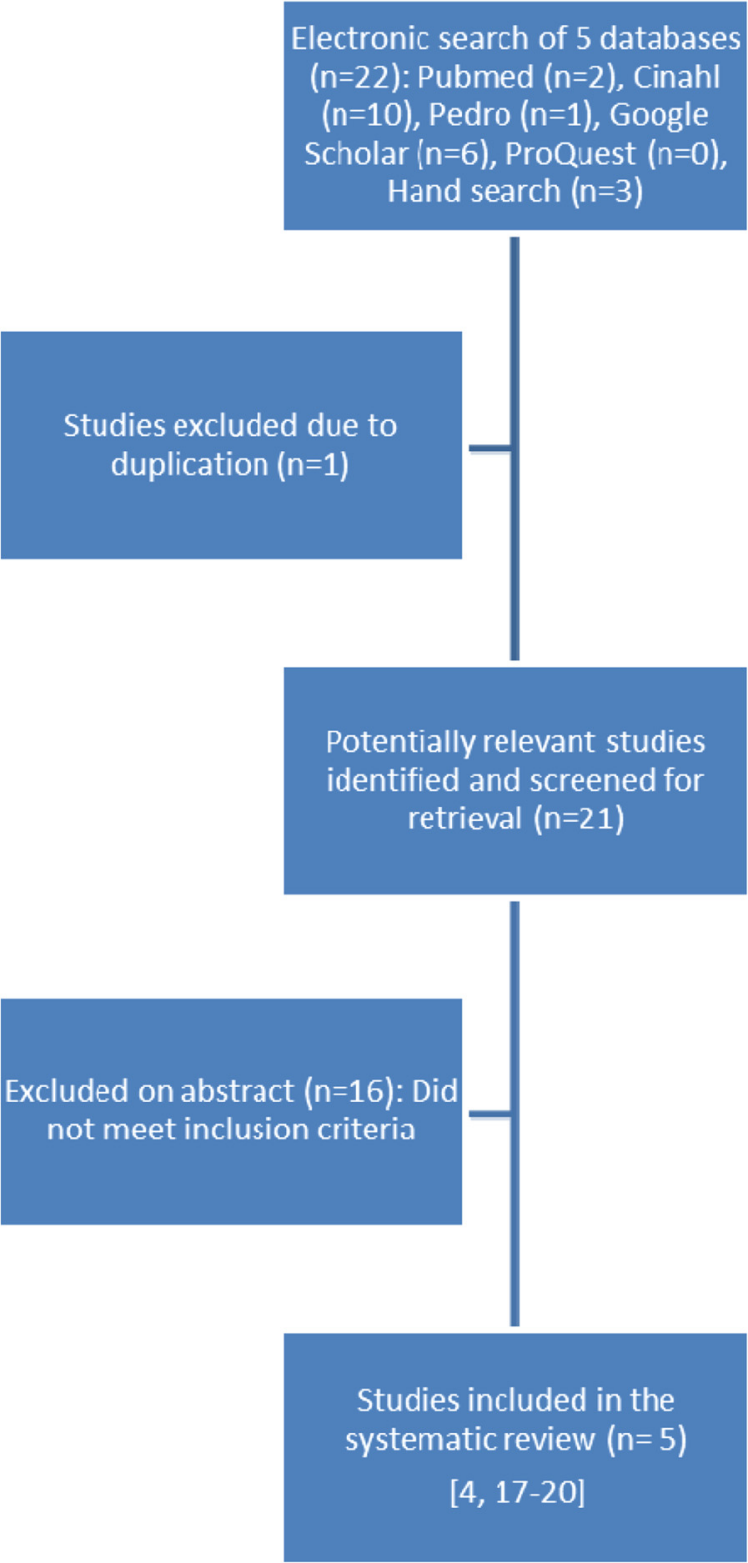
Selection of studies: summary of studies in order of level of evidence, with extracted data.

The computer-generated search resulted in a potential 2 references in Pubmed, 10 in Cinahl, 1 in Pedro, 6 in Google Scholar and 0 in ProQuest. Pearling of reference lists of relevant articles produced 3 new articles which matched the inclusion criteria. After exclusion of the duplicated references, both reviewers (SvN and SH) read 18 titles and abstracts. The most frequent reasons for exclusion were: studies did not have pre/post measurement and studies did not isolate the chair as an intervention. Finally 5 studies were included in this review [4,17-20] (Figure 1).

#### Risk of bias assessment

**Figure 2:**
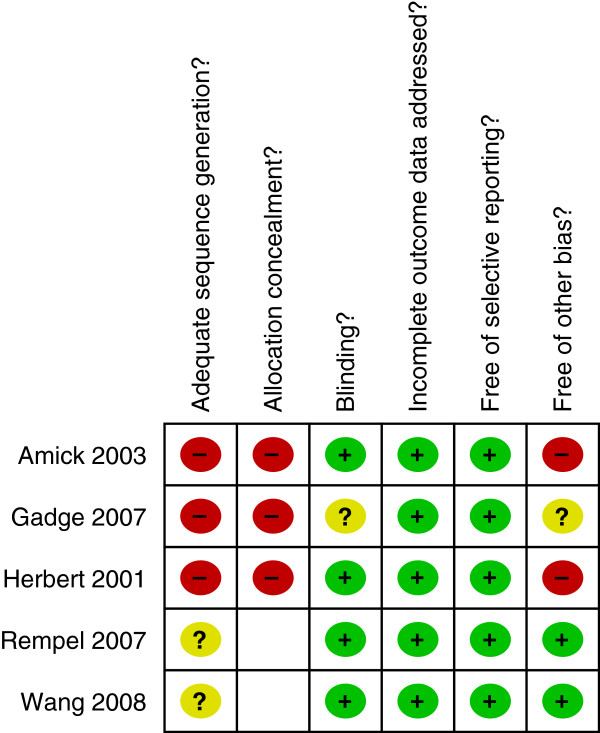
Methodological quality summary: review authors' judgements about each methodological quality item for each included study (blank spaces or ‘?’ denote criterion not able to be determined or unclear).

Overall there was a moderate risk of bias evaluated for the body of evidence. The most likely source of bias was in allocation concealment and generation of the random sequence. The areas in Figure 2 marked with a ‘?’ or a blank space indicate that the reviewers were not able to determine whether the criterion was met.

### Study characteristics

Table 1 shows the characteristics of the studies included in the systematic review. The number of participants varied from 4 to 293 participants. Three of the five studies were RCTs [4,17,19], one was a pre/post-test intervention study [20] and one single case, multiple baseline (ABAB) study was reported [18]. Three studies were conducted in a garment factory [17,19,20], one in an office environment [4] and one with university students [18]. *Two of the papers included were from the same funded trial (Los Angeles Garment Study) but reported on different subgroups: Wang et al*[17]*reported on symptom change in the garment worker subject group with initial pain/discomfort in the low back/hip regions and Rempel et al*[19]*reported on the sub group with pre-intervention cervical/shoulder symptoms. The two groups may have had some overlap but this was not reported in detail.*

**Table 1:** Selected studies: summary of studies in order of level of evidence, with extracted data

**Author (ref)**	**Country**	**Design**	**n**	**Population**	**Intervention**	**Measures**	**Outcomes**	**Conclusion**	**Notes**
Wang et al. 2008 [17]	USA	RCT	293 (subset of operators with Rempel 2007 with lower p) Group n= (111;84;98)	Sewing machine operators with back /hip pain	Gp 1: control	Pre and post monthly for 4/12: Pain symptoms- intensity (1-5) and frequency	Mean pain Improv’t gp 2 vs 1: 0.25(95%CI: 0.16, 0.34);GP3 vs 1: 0.43 (0.34-0.51) per month.	Adjustable, swivelling chairs offer advantage (reduction in LB/Hip pain) for workers in seated/UL occupations; flat pan superior to curved?	Obtain means and sd for pain scores for each group (presented graphically in Fig 5A) at 4/12f/u
					Gp2: curved pan chair				
					Gp 3: flat seat pan chair (all received misc items, chairs hgt adjustable)				
Remple et al. 2008 [19]	USA	RCT	277 (subset with upper p) Group n pain (105;72;100)	Sewing machine operators with neck/ shoulder pain	Gp 1: control Gp2: curved pan chair Gp 3: flat seat pan chair (all received miscel items; intervention chairs hgt adjustable)	Pre and post monthly for 4/12: Pain symptoms- intensity (1-5) and frequency	Mean pain Improv’t gp 2 vs 1: 0.34 (95% CI: 0.28, 0.41); GP3 vs 1: 0.14 (.07-.022) per month.	Adjustable, swivelling chairs offer advantage (reduction in Cx/shoulder pain) for workers in seated/ UL occupations; curved pan superior to flat?	Obtain means and sd for pain scores for each group (presented graphically in Fig 5A) at 4/12f/u
Amick et al. 2003 [4]	USA	RCT (assigned according to office location)	192(87;52;S3)	Office workers (>4hrs per day at computer;>6 hrs per day sitting)	Gp1: adjustable chair + training	Pre (2xmonthly) and post intervention (3x over 1 year). Musculo- skeletal symptoms-1. Growth over workday 2. Average pain over workday	Symptom growth over workday: Gp 1<gp2/3 at 12/12f/u (p=0.012). Ave pain levels: Reduced for both Gp 1+2 compared to Gp3	Highly adjustable chairs plus training resulted in less end of day pain and reduced average pain (largest reduction in neck/shoulder, followed by upper and lower back)	Cant separate chair as sole intervention but clear that chair + info is superior to info alone or nothing.
					Gp2: training only				
					Gp3: no intervention				
Herbert et al. 2001 [20	USA	Pre and post test	36	Garment workers (“spooling” task), female	Adjustable chairs and training in their use	MS symptom survey prior to and 6/12 after introduction. Joint position in sitting via video (subgroup only). Upper limbs only.	Baseline pain report89% of group; post 63.9% (p=0.007); Reduction in severity at 10/11 anatomic sites after intervention. Only modest declines in awkward posture (small n)	Reduction in people with pain and reduction in severity overall at upper limb anatomical sites. Inconclusive posture change findings.	
Gadge et al. 2007 [18]	Australia	Sungle case, multiple baseline (ABAB)	4	University students (sitting “most of the time”)	Standard office chair (adjustable) vs “saddle” seat	(dis) Comfort (VAS); Production (typing task speed and accuracy); Posture (videotape) Multiple measures across 4 phases.	Discomfort in lower back increased over time in both chairs but less so in the saddle; discomfort was significantly worse in lower limbs in saddle chair; Productivity no change; Greater trunk to thigh angles in saddle.	Some benefits for lower back discomfort and posture in saddle but also issues (lower limb discomfort).	

#### Study outcomes

All five studies found a reduction in self-reported musculoskeletal pain or discomfort after the intervention [4,17-20]. The most common feature of the chair intervention itself was that it was adjustable (all five studies). There were variations added to this primary quality including curved pan versus flat seat [17,19], or saddle seat [18]. Training in the use of the adjustable features was also prominent in all studies. The body sites for decreased pain were different for each study: one reported back/hip pain [17]; one neck/shoulder pain [19]; one musculo-skeletal symptoms anywhere [4] but reported the greatest reduction in pain was in the neck/shoulder followed by upper and lower back; one upper limb only [20] and the final paper reported on lumbar spine discomfort [19]. Only one study investigated productivity outcomes [18] and found no significant differences. Similarly the two studies that assessed elements of posture (for example thigh angles) also found modest to no differences with their chair interventions [18,20].

### Data analysis

It was not possible to perform a meta-analysis because of the clinical heterogeneity of the trials. *The sources of this heterogeneity included differing populations, interventions and outcomes. These were all different from one study to another, with one exception: Rempel et al and Wang et al had the same intervention and overall population but reported different subgroups and outcomes*[17,19]*.* As such sensitivity analysis was also not able to be performed.

## Discussion

We found five studies of moderate quality that offer some support for the use of chair interventions to improve musculo-skeletal pain or discomfort in workers who sit for prolonged periods. However there was a high degree of clinical heterogeneity meaning that more specific conclusions cannot be drawn. Because of the high occurrence of musculoskeletal problems among office workers, changes to their chairs are often recommended. The shortage of evidence involving office workers (only one study) is thus of concern, considering the investment in ergonomic chairs by corporations and companies. There is also a lack of evidence to assess the effect of chairs on children and adolescents in preventing or reducing musculoskeletal symptoms. Further research into this population with growing keyboard time is required - reinforced by the increasing trend of musculoskeletal symptoms among youths [21-23].

The findings of this review indicate a consistent trend of support for the role of a chair intervention to reduce the severity, intensity and frequency of musculoskeletal pain among workers who are required to sit for prolonged periods. However because the studies reported different body areas it is not possible to be more specific about which kinds of musculoskeletal pain benefit the most. The most common parameter introduced in the chair intervention/s was to have an adjustable feature such as seat and back height. Electromyographic (EMG) studies have reported that a chair which is height adjustable and has adjustable backrest and armrests can reduce the muscle activity of the neck, shoulder and back, and also decreases the inter-vertebral disc pressure [24-26]. Therefore there is some support that adjustability of the chair can be directly associated with the function of the musculoskeletal system. The second most common feature reported as a chair intervention, was that the participants received training in the use of their chair (how to adjust appropriately). This is intuitive and it is now valuable to have studies which support this as an essential feature of ergonomic interventions.

Other features of the interventions varied such as curved pan versus flat seating – two *studies*[17,19]*compared these with* some suggestion that curved pan seating may be better in reducing upper body pain whilst flat seating may be superior for lower body pain. The authors postulated that the *curved, 2-part seat pan supports the forward leaning posture by allowing a more open thigh-torso angle.* These findings need confirmation in further studies. Saddle seating also seemed to have differential effects on back versus lower limb comfort – again this requires further careful investigation before recommendations can be made [18].

The study by Gadge [18] was the only eligible publication which included productivity as an outcome. The study sample was very small and the types of outcomes – i.e. typing speed and errors – were not relevant to all seated workers. It is an assumption that ergonomic intervention correlates with productivity [9,27]. However, this review found no supporting evidence for positive gains in productivity and this factor should be incorporated as an outcome in future research. No studies reported on cost aspects of the intervention.

Although all five studies conducted follow-up assessments of the symptoms, the longest follow-up period was only a year [4]. This indicates a gap in showing whether the effectiveness of a chair intervention has long-term benefits, particularly with respect to musculoskeletal symptoms, as well as the recurrence of symptoms and the consequent cost of care. Chronicity in work-related musculoskeletal pain is multifactorial, with risk profiles relating to psychosocial factors dominating the literature [28]. We believe future studies, addressing long-term effects, need to be designed to take these factors into account.

The effect of bias on the interpretation and trustworthiness of the evidence cautions against making conclusive recommendations pertaining to the effect of a chair intervention. The key methodological shortcomings which introduced bias were absent/unclear randomization procedures and concealed allocation. These may introduce selection bias which can result in a higher association (odds ratio) between the exposure and the subject. Because of the occurrence of selection bias, it is also not possible to relate the results to the general population. *A further methodological issue arose in that two papers used the same overall population to report two different subgroups (based on two regions of pain). We therefore treated these sub-groups as two studies, assuming pain regions were independent events.* Across the board the authors of the reviewed articles failed to mention whether confounding factors, such as female gender, were controlled for as the allocation procedures were not mentioned. Future research should address these methodological shortcomings to improve the validity of the findings and thereby increase the quality of the evidence to support a chair intervention.

### Recommendations

Clinical implications - clinicians can cautiously support or advocate for the provision of adjustable chairs in the workplace and offer appropriate training in how to adjust and manage posture whilst seated. Monitoring of pain reduction/increased comfort ratings will confirm effectiveness in individual cases.

Research implications – further urgent research is required to clarify the relationship between environmental features (such as chairs), poor posture and symptoms as currently these relationships are inferred. Furthermore specific effectiveness research is required to confirm the reviewed studies using

Clearly defined interventions;

Outcome measures that include symptoms as well as performance;

Cost-effectiveness needs to be measured to allow interpretation of health benefits in light of intervention costs;

Longer term follow-up to monitor effects after the period of observation/attention;

Robust methodology (in particular concealed allocation and randomisation);

Other populations including occupational groups in the information technology and call centre industries, adolescents and children who are also required to sit for prolonged periods.

## Conclusion

The findings of this review indicate a consistent trend of support for the role of a chair intervention to improve musculoskeletal symptoms among workers who are required to sit for prolonged periods. The small number of studies and moderate risk of bias impacts on the interpretation and strength of the evidence. We can make cautious recommendations pertaining to the effect of a chair intervention – in particular that adjustable chairs with appropriate training hold the most promise. We have identified gaps in showing whether the effectiveness of a chair intervention has long-term benefit, particularly with respect to musculoskeletal symptoms as well as the recurrence of symptoms and the consequent cost of care.

## Competing interests

The authors confirm that there is no competing interests, real or perceived.

## Authors’ contributions

SvN conceived of the study, carried out the literature search, and drafted the manuscript. QL participated in the design of the study and helped to draft the manuscript. SH performed the data extraction, analysis and participated in its design. All authors read and approved the final manuscript.

## Pre-publication history

The pre-publication history for this paper can be accessed here:

http://www.biomedcentral.com/1471-2474/13/145/prepub
